# Preterm birth and reduced birthweight in first and second teenage pregnancies: a register-based cohort study

**DOI:** 10.1186/1471-2393-10-36

**Published:** 2010-07-09

**Authors:** Ali S Khashan, Philip N Baker, Louise C Kenny

**Affiliations:** 1 Anu Research Centre, Department of Obstetrics and Gynaecology, University College Cork, Cork, Ireland; 2 The Maternal and Fetal Health Research Centre, University of Manchester, Manchester, UK

## Abstract

**Background:**

Higher risks of preterm birth and small for gestational age babies have been reported in teenagers. The aim of this study was to investigate the relationship between first and second teenage pregnancies and preterm birth, birthweight and small for gestational age (SGA).

**Methods:**

All women aged 14 to 29 yrs who gave birth to live singletons in the North Western Region of England between January 1st 2004 and December 31st 2006 were identified. Women were classified in three groups; 14-17 yrs, 18-19 yrs and 20-29 yrs (reference group). The outcome measures were preterm birth, very preterm birth, birthweight, SGA (< 5^th ^percentile), very SGA (VSGA< 3^rd ^percentile). We compared these outcome measures in teenagers' first and second pregnancies with those of mothers aged 20 to 29 yrs.

**Results:**

The risk of preterm birth was increased in first (OR = 1.21, [95% CI: 1.01-1.45]) and second (OR = 1.93, [95% CI: 1.38-2.69]) time mothers aged 14-17 yrs compared to the reference group. Birthweight was reduced in the first (mean difference = -24 g; [95% CI: -40, -7]) and second (mean difference = -80 g; [95% CI: -115, -46]) time mothers aged 14-17 yrs compared to the reference group. There was some evidence of a protective effect against VSGA in 14-17 yr old first time mothers (OR = 0.79, [95% CI: 0.63-0.99]).

**Conclusions:**

Teenage mothers are at increased risk of preterm birth compared to adult mothers and this risk is further increased in second time teen pregnancies. This study highlights the importance of ensuring pregnant teenagers have appropriate antenatal care. A first pregnancy may be the first and only time a pregnant teenager interacts with health services and this opportunity for health education and the promotion of contraception should not be overlooked.

## Background

The United Kingdom is widely quoted as having the highest teenage pregnancy rate in Western Europe[1,2] and this has barely altered in the last three decades[3]. In England and Wales, the rate of conceptions per 1000 women aged under 20 was 60.0 in 2007 and 61.7 in 2008[4]. In 1998, the teenage birth in the United Kingdom was 30.8 compared to 9.3 in France, 14 in Germany and 7.9 in Spain[5]. Several studies reported an association between teenage pregnancy and increased risks of preterm birth,[6,7] neonatal mortality[8,9], congenital anomalies[10] and low birthweight[11] although others found no association[12,13].

The association between young maternal age and adverse pregnancy outcome has been attributed to gynaecological immaturity and the growth and nutritional status of the mother[14]. The reduction in fetal growth described in some studies has been proposed to result from competition for nutrients between the still growing adolescent mother and her fetus. However, this theory is controversial[11,15] as there is also a strong association between teenage pregnancy and socioeconomic deprivation and teenage mothers are more likely to smoke, drink alcohol and have poor diet[2,16,17]. These factors alone or in combination may also influence pregnancy outcome.

Most studies investigating teenage pregnancy compare outcomes in teenagers with outcomes in adult mothers. Many investigators were unable to control for the combination of adverse socioeconomic pressures[18,19]. Furthermore, 25% of all UK first time pregnant teenagers become pregnant again in their teenage years[20]. Several studies have investigated the effect of parity on adverse pregnancy outcome [2,16,17,21], however, the results are inconsistent. For example, Olausson et al[16] suggested that teenage mothers are at higher risk of preterm birth in their first pregnancy compared to teenage mothers having their second baby and compared to adult women. In contrast, Smith and Pell[2] found that first teenage pregnancies are not associated with adverse pregnancy outcomes at all but second teenage pregnancies are associated with higher risk of preterm birth and stillbirth.

The North West of England is well placed to study the outcomes of teenage pregnancy. The North Western Perinatal Survey collects routine maternal, obstetric and neonatal information on all infants delivered in the region's 21 maternity hospitals. These hospitals cover a wide geographic area containing a mixture of urban and rural communities and provide for women from a variety of ethnic groups and socio-economic strata, including those residing in areas of considerable social deprivation.

The aims of this study were to determine whether teenage pregnancy was associated with reduced infant birthweight and with increased rates of small for gestational age (SGA) and preterm birth, whether the association differed by parity, and whether any associations were independent of confounding factors.

## Methods

We performed a population based cohort study using a database generated from the North Western Perinatal Survey (NWPS), based at St Mary's Hospital, Manchester, UK. We identified all singleton live infants who were born between January 1, 2004 and December 31, 2006. The data were restricted to women aged between 14 and 29 yrs in their first or second pregnancies.

We defined first births as the first pregnancy to result in a singleton live born infant and second births were defined as the second pregnancy to result in a singleton live born infant. Women were classified into 3 groups according to their age at the time of delivery; 14-17 yrs, 18-19 yrs and 20-29 yrs of age.

Birthweight records were considered invalid if they were less than 500 g or more than 5500 g. Preterm delivery was defined as birth ≥ 33 and < 37 gestation weeks and very preterm delivery was defined as birth ≥ 23 and < 33 weeks. We used individualised birthweight ratios (IBR) to generate a measure for SGA. IBR corrects birthweight for gestational age taking into account ethnic origin, infant sex, parity and maternal height and weight. The IBR provides a better indicator of perinatal mortality than birthweight for gestational age alone[22]. Babies were considered SGA if their IBR was below the 5^th ^percentile and very SGA (VSGA) if their IBR was below the 3^rd ^percentile.

### Statistical Analysis

For the statistical analysis we used multiple regression models which allow us to control for the potential confounding effect of several variables on the association between teenage pregnancy and the outcome measures. Multiple logistic regression was used to estimate the odds ratio (OR) of preterm birth, very preterm birth, SGA and VSGA. All ORs were adjusted for maternal ethnicity, body mass index (BMI) and infant sex using the categories indicated in Table 1. We also adjusted for a social deprivation score determined by post-code which is based on seven deprivation domains: income, employment, health and disability, education, barriers to housing and services, living deprivation and crime[23]. We categorised women into four quartiles based on their social deprivation score and each quartile consisted of approximately 22.5% of the population. The rest of the women (9.2%) were not linked to the social deprivation score because their post-codes were missing; these women were categorised separately in a fifth group.

**Table 1:** Maternal demographic characteristics in relation to age group and parity

	Women aged 14-17		Women aged 18-19		Women aged 20-29	
						
	First births (n = 3065) n(%)	Second births (n = 571) n(%)	First births (n = 5316) n(%)	Second births (n = 2190) n(%)	First births (n = 22710) n(%)	Second births (n = 22501) n(%)
**Social deprivation score**						
1 (least deprived)	242 (7.88)	31 (5.43)	532 (10.01)	134 (6.12)	4406 (19.40)	3582 (15.92)
2	587 (19.12)	107 (18.74)	1043 (19.62)	371 (16.94)	5553 (24.45)	4907 (21.81)
3	844 (27.49)	158 (27.67)	1517 (28.54)	610 (27.85)	5357 (23.59)	5798 (25.77)
4 (most deprived)	1062 (34.59)	215 (37.65)	1634 (30.74)	847 (38.68)	5095 (22.44)	6051 (26.89)
unclassified	335 (10.91)	60 (10.51)	590 (11.10)	228 (10.41)	2299 (10.12)	2163 (9.61)
**Ethnicity**						
White	2520 (82.08)	479 (83.89)	4087 (76.88)	1767 (80.68)	15218 (67.01)	15455 (68.69)
Asian	55 (1.79)	12 (2.10)	286 (5.38)	93 (4.25)	2394 (10.54)	2634 (11.71)
Indian	8 (0.26)	1 (0.18)	71 (1.34)	22 (1.00)	889 (3.91)	721 (3.20)
Black	90 (2.93)	11 (1.93)	115 (2.16)	57 (2.60)	683 (3.01)	642 (2.85)
Chinese	4 (0.13)	2 (0.35)	11 (0.21)	5 (0.23)	145 (0.64)	96 (0.43)
Other and not recorded	393 (12.80)	66 (11.56)	746 (14.03)	246 (11.23)	3381 (14.89)	2953 (13.12)
**BMI**						
Normal (18.5-24.9)	1381 (44.98)	248 (43.43)	2196 (41.31)	862 (39.36)	8463 (37.27)	7640 (33.95)
Underweight (12-18.4)	144 (4.69)	15 (2.63)	229 (4.31)	81 (3.70)	559 (2.46)	547 (2.43)
Overweight (25-29.9)	359 (11.69)	88 (15.41)	757 (14.24)	341 (15.57)	3830 (16.86)	3990 (17.73)
Obese (30-39.9)	116 (3.78)	28 (4.90)	309 (5.81)	160 (7.31)	2028 (8.93)	2453 (10.90)
Morbidly obese (> 40)	9 (0.29)	1 (0.18)	23 (0.43)	18 (0.82)	207 (0.91)	307 (1.36)
Missing	1061 (34.56)	191 (33.45)	1802 (33.90)	728 (33.24)	7623 (33.57)	7564 (33.62)

We used linear regression with robust estimation variance to estimate the difference in mean birthweight among teenage mother groups compared with adult women. The models were adjusted for social deprivation score, parity, BMI, ethnicity and gestational age. Since the relationship between birthweight and gestational is not linear, we used fractional polynomial regression to determine the best functional model which resulted in adding four fractional polynomial functions of gestational age to the model. The teenage group was divided into two groups: 14-17 and 18-19 yrs of age. We compared the outcome measures of women in each of the teenage groups with those of the adult women (reference group). All models were carried out separately for first and second births. All statistical analyses were performed using Stata Software 10.

### Smoking confounding effect

From 2007, additional information regarding maternal smoking at the first antenatal visit has been recorded by the NWPS. Thus we had access to data on all 2007 births from the NWPS in which to explore the potential confounding effect of maternal smoking on the association between young maternal age and preterm birth and birthweight. We carried out two sets of models; the first set were adjusted for social deprivation score, BMI, ethnicity and infant sex while the second set were adjusted for the same variables in addition to maternal smoking. These models were carried out separately for primiparous and multiparous patients.

The present study used anonymised data and it is not possible to identify participants. The director of the NWPS gave permission to conduct this study. Thus ethical approval was neither required nor requested.

## Results

The cohort consisted of mothers of 56,353 singleton live babies. Mothers of 3,636 babies were between 14-17 yrs of age at the time of birth, mothers of 7506 babies were 18-19 yrs and mothers of 45,211 babies were 20-29 yrs old. Table 1 presents the distribution of the study population across maternal age groups and first and second pregnancies. There were missing data on social deprivation score, ethnicity and BMI. However, the missing data in each of these variables were comparable across the subgroups. In primiparous women, mean birthweight was 3,271 g in adults, 3,231 g in 18-19 yrs old and 3,220 g in 14-17 yrs old. In second time mothers, mean birthweight was 3,326 g in adults, 3,248 g in 18-19 yrs old and 3,168 g in 14-17 yrs old. The rates of teenage pregnancy in both the 14-17 yrs old group and 18-19 yrs old group increased with increasing social deprivation such that more than one third of the teenage mothers came from the most socially deprived areas. Moreover, second time teenage mothers (14-17 and 18-19) were more likely than all other women in the study to come from the most socially deprived areas. Teenage mothers were more likely to be underweight and of white ethnic background.

### First births: 14-17 vs. 20-29 yrs old mothers

The adjusted analyses suggested that teenagers aged 14-17 had a significantly higher risk of preterm birth (adjusted OR = 1.21, [95% CI: 1.01-1.45]) and very preterm birth (adjusted OR = 1.71, [95% CI: 1.29-2.26]) compared to adult women (Table 2). Mean birthweight was significantly reduced by 24 grams in first time teenage mothers aged 14-17 yrs (adjusted difference = -24 g; [95% CI: -40, -7]). Similar results were obtained after restricting the analysis to term babies. However, when birthweight was examined using IBRs we found that teenagers who gave birth for the first time at 14-17 yrs of age were at significantly lower risk of VSGA (adjusted OR = 0.79, [95% CI: 0.63-0.99]) and a non-significant lower risk of SGA (adjusted OR = 0.89, [95% CI: 0.74-1.07]).

**Table 2:** Estimates of outcome measures among women aged 14 to 17 years compared with women aged 20 to 29 years

	First Births (n = 25780)			Second births (n = 23072)		
						
Outcomes	Number of babies	Crude estimate^a ^(95% CI)	Adjusted estimate^a ^(95% CI)	Number of babies	Crude estimate^a ^(95% CI)	Adjusted estimate^a ^(95% CI)
Birthweight in grams	3065	-51(-73, -29)	-24(-40, -7)**^b^**	571	-158(-209, -107)	-80(-115, -46)**^b^**
Birthweight in term babies	2847	-39(-59, -19)	-27(-44, -11)**^b^**	512	-120(-166, -74)	-88(-124, -52)**^b^**
SGA	147	0.88(0.73-1.05)	0.89(0.74-1.07)**^c^**	42	1.33(0.96-1.85)	1.23(0.88-1.71)**^c^**
VSGA	88	0.78(0.62-0.98)	0.79(0.63-0.99)**^c^**	32	1.49(1.03-2.16)	1.38(0.95-2.00)**^c^**
Preterm birth 33-36 weeks	151	1.23(1.03-1.48)	1.21(1.01-1.45)**^c^**	40	1.87(1.33-2.65)	1.93(1.38-2.69)**^c^**
Very preterm birth 23-32 weeks	65	1.45(1.07-1.95)	1.71(1.29-2.26)**^c^**	15	2.06(1.20-3.55)	1.87(1.10-3.18)**^c^**

a The estimate of the effect of young maternal age on birthweight is birthweight mean difference in grams; the estimates of SGA, VSGA, preterm birth and very preterm birth are odds ratios.

b adjusted for BMI, social deprivation score, infants sex, ethnicity and gestational age.

c adjusted for BMI, social deprivation score, infants sex and ethnicity

### Second births: 14-17 vs. 20-29 yrs old mothers

The ORs of preterm birth (adjusted OR = 1.93, [95% CI: 1.38-2.69]) and very preterm birth (adjusted OR = 1.87, [95% CI: 1.10-3.18]) were increased significantly in second time mothers aged 14-17 yrs compared with second time adult mothers (Table 2). Women aged 14-17 yrs at the time of their second delivery had babies with significantly smaller birthweight compared to second births in adult women (adjusted difference = -80 g; [95% CI: -115, -46]). The estimate was similar after restricting the analysis to term babies. Among second births in the 14-17 yrs old age group, there was a non-significant increase in risk of SGA (adjusted OR = 1.23, [95% CI: 0.88-1.71]) and VSGA (adjusted OR = 1.38, [95% CI: 0.95-2.00]).

### First birth: 18-19 vs. 20-29 yrs old mothers

The adjusted analyses suggested that 18-19 yr old primiparous women were at a significantly increased risk of very preterm birth (adjusted OR = 1.42, [95% CI: 1.12-1.81]) but not preterm birth (adjusted OR = 1.10, [95% CI: 0.95-1.28]) (Table 3). In addition, mean birthweight was significantly reduced by 29 grams in 18-19 yrs old primiparous women (adjusted difference = -29 g; [95% CI: -42, -16]) compared with adult primiparous women. The estimate was similar when the analysis was restricted to term babies. Among 18-19 yrs old primiparous women there was no significant increase in the risk of SGA (adjusted OR = 1.13, [95% CI: 0.99-1.28]) or VSGA (adjusted OR = 1.09, [95% CI: 0.93-1.27]) compared with 20-29 yrs old primiparous women.

**Table 3:** Estimates of outcome measures among women aged 18 to 19 years compared with women aged 20 to 29 years

	First Births (n = 28026)			Second births (n = 24691)		
						
Outcomes	Number of babies	Crude estimate^a ^(95% CI)	Adjusted estimate^a ^(95% CI)	Number of babies	Crude estimate^a ^(95% CI)	Adjusted estimate^a ^(95% CI)
Birthweight in grams	5316	-40(-57, -23)	-29(-42, -16)**^b^**	2910	-78(-103, -53)	-55(-74, -37)**^b^**
Birthweight in term babies	4969	-33(-49, -16)	-30(-43, -16)**^b^**	2044	-71(-95, -48)	-57(-77, -38)**^b^**
SGA	325	1.13(0.99-1.28)	1.13(0.99-1.28)**^c^**	165	1.35(1.13-1.60)	1.25(1.05-1.48)**^c^**
VSGA	212	1.09(0.94-1.28)	1.09(0.93-1.27)**^c^**	107	1.27(1.03-1.06)	1.17(0.94-1.45)**^c^**
Preterm birth 33-36 weeks	240	1.07(0.92-1.25)	1.10(0.95-1.28)**^c^**	106	1.43(1.16-1.76)	1.27(1.03-1.59)**^c^**
Very preterm birth 23-32 weeks	94	1.45(1.14-1.84)	1.42(1.12-1.81)**^c^**	38	1.22(0.84-1.76)	1.21(0.86-1.71)**^c^**

a The estimate of the effect of young maternal age on birthweight is birthweight mean difference in grams; the estimates of SGA, VSGA, preterm birth and very preterm birth are odds ratios.

b adjusted for BMI, social deprivation score, infants sex, ethnicity and gestational age.

c adjusted for BMI, social deprivation score, infants sex and ethnicity

### Second birth: 18-19 vs. 20-29 yrs old mothers

The estimates suggested that mean birthweight was reduced by 55 grams in second time mothers aged 18-19 yrs (adjusted difference = -55 g; [95% CI: -74, -37]) compared with second time adult mothers and the result was similar when the analysis was restricted to term babies. These women were at significantly increased risk of SGA (adjusted OR = 1.25, [95% CI: 1.05-1.48]) and preterm birth (adjusted OR = 1.27, [95% CI: 1.03-1.59]) but not VSGA (adjusted OR = 1.17, [95% CI: 0.94-1.45]) and very preterm birth (adjusted OR = 1.21, [95% CI: 0.86-1.71]) compared with second time adult mothers (Table 3).

### Smoking confounding effect

The 2007 dataset consisted of 55,539 singleton live births. The final cohort consisted of 30,360 babies of mothers aged 14-29 yrs. Mothers of 930 babies were 14-17 yrs of age, 2,792 were aged 18-19 yrs and 26,638 women were 20-29 yrs. 7294 were smokers, 21,056 were non-smokers and 2010 had missing smoking status. The estimates of the effect of young maternal age on birthweight and preterm birth with and without adjustment for smoking are presented in Table 4. These results suggest that maternal smoking has little confounding effect on the association between young maternal age in primiparous and multiparous women and preterm birth. However, the association between young maternal age and birthweight could be partly related to the confounding effect of smoking.

**Table 4:** Smoking confounding effect on the estimates of birthweight and preterm birth in the 2007 dataset

	Primiparous				Multiparous			
	**14-17 years**		**18-19 years**		**14-17 years**		**18-19 years**	

**Outcomes**	**Not adjusted for smoking^a^**	**Adjusted for smoking^b^**	**Not adjusted for smoking^a^**	**Adjusted for smoking^b^**	**Not adjusted for smoking^a^**	**Adjusted for smoking^b^**	**Not adjusted for smoking^a^**	**Adjusted for smoking^b^**

Birthweight estimates in grams	-28(-59, 3)	4(-27, 35)	-37(-58, -15)	-11(-32, 11)	-77(-137, -16)	-72(-132, -13)	-56(-83, -29)	-34(-61, -6)
Preterm birth OR	1.09(0.59, 2.02)	1.08(0.58, 2.00)	1.08(0.79, 1.47)	1.03(0.76, 1.41)	1.85(1.34, 2.56)	1.76(1.27, 2.44)	1.36(1.07, 1.73)	1.29(1.01, 1.65)

a adjusted for the same variables as in Tables 2 & 3.

b adjusted as in a + maternal smoking

## Discussion

Compared with older women, women who gave birth during the teenage years were at increased risk of preterm and very preterm delivery. This risk was higher for younger teenager mothers (14-17 yrs) than for older teenagers (18-19 yrs) and in the 14-17 yrs old group the risk was greater in second pregnancies than in first. Our findings are in contrast to a recent Swedish longitudinal study which reported that teenagers were at a higher risk of preterm birth in their first as compared to their second births[16]. Previous longitudinal studies of first and second birth among teenagers have produced conflicting results [21,24,25]. The weakness of longitudinal studies is that generally first births are at greater risk of preterm birth (and other pregnancy complications) than subsequent births[26,27]. The cross sectional design of our study allowed the normal protective effect of second birth to be taken into account. Indeed, in a similar cross sectional study from the UK, Smith and Pell[2] described an increased risk of both preterm and very preterm birth in teenage women and this risk was greatest for second births. Our results are also consistent with an American study which reported an increased risk of preterm birth in teenage women for second births[17].

Although we found a significant reduction in birthweight of babies born to teenage mothers compared to adult mothers, when individualised birthweight ratios (IBRs) were used, teenagers having their first baby did not have an increased risk of delivering either SGA and VSGA babies. In teenagers having their second baby, the risks of SGA and VSGA were not significantly increased apart from SGA in teenage women aged 18-19 yrs. These findings are in contrast to an American study which found that teenage birth was independently associated with risk of intrauterine growth restriction[7]. However, Frazer et al[7] failed to adequately control for social deprivation. They classified teenagers into socioeconomic groups based on marital status, education level attainment, and the level of prenatal care received. These factors may not be truly representative of adverse socioeconomic pressures. Although not perfect, we used deprivation scores based on postcode sector; these scores have been shown to be strongly associated with deprivation related diseases[28]. Secondly, Frazer et al., defined small-for-gestational-age infants as those with birth weights below the 10th percentile for gestational age and sex. Unlike our use of IBRs, this does not take into account the additional effects of maternal height, weight and ethnicity on birthweight. Indeed, when outcomes were compared with the age range 20-29 yrs, women aged 14-17 yrs had a decreased risk of VSGA, which is consistent with a more recent Scottish study [2] and with a previous population based study from the United States [29].

Although our study was population-based, there were limitations. Firstly, there were missing data on the potential confounders, although the missing data appeared to be equally distributed across the maternal age groups. Therefore these missing data are unlikely to have affected our reported estimates. Secondly, the main cohort lacked information on maternal smoking. The 2007 data suggested that although maternal smoking had some effect on the association between young maternal age and birthweight, there was little if any confounding effect on the association between young maternal age and preterm birth. However, maternal smoking data are often subject to misclassification as a substantial proportion of pregnant smokers misreport their smoking status. Moreover many quitters are reported to smoke again during pregnancy[30,31]. England et al., reported that an estimated 24% of active smokers were misclassified as quitters[31]. It is therefore possible that there is still an unmeasured smoking confounding effect in the present study due to misclassification of maternal smoking status.

It is unlikely that the association between second teenage pregnancy and preterm birth can be explained by differences in the interval between pregnancies among teenage and older mothers since the associations observed were much greater than those previously reported for short intervals between pregnancies[32]. It is possible that the increased risk of poor pregnancy outcome is related to biological immaturity. It is also possible that the increased risk of poor pregnancy outcome in the second teenage pregnancy is related to less prenatal care in the second pregnancy than in the first. Teenage pregnant women, mainly those having second teenage pregnancy, are less likely to seek prenatal care than adult pregnant women [15,33,34].

## Conclusions

In conclusion, our findings suggest an association between second teenage delivery and preterm birth and birthweight independent of maternal social deprivation, ethnicity, BMI and smoking. Teenagers have an increased risk of preterm and very preterm delivery and this risk is greatest for younger teenagers and those in their second pregnancy. In contrast to previous studies, there was little evidence for an association between teenage pregnancy and risk of delivering a small for gestational age infants. This study highlights the importance of ensuring pregnant teenagers have appropriate antenatal care. Moreover a vital component of this care is post-natal contraception to prevent a second teenage pregnancy with potentially higher risks of adverse outcomes. A first pregnancy may be the first and only time a pregnant teenager interacts with health services and this opportunity for health education and the promotion of contraception should not be overlooked.

## Competing interests

The authors declare that they have no competing interests.

## Authors' contributions

all co-authors contributed to the 1) conception and design or analysis and interpretation of the data; 2) drafting the article or revising it critically for important intellectual content; 3) all co-authors approved the submission of this version of the manuscript.

## Pre-publication history

The pre-publication history for this paper can be accessed here:

http://www.biomedcentral.com/1471-2393/10/36/prepub
